# Enhancing Participant Recruitment and Retention in Dementia Research through Community Engagement

**DOI:** 10.1002/alz70856_105505

**Published:** 2026-01-08

**Authors:** Michelle Nichols

**Affiliations:** ^1^ University of South Carolina, Charleston, SC, USA

## Abstract

**Background:**

Participant recruitment and retention are major concerns for research teams and can influence study success and relevance of findings. Known challenges include access to relevant populations, mistrust, fear, lack of awareness of research, sociocultural considerations, complexity of studies, burden, literacy, and language barriers.

Community Engaged (CE) Research builds sustainable community‐researcher partnerships to advance science through co‐ownership and co‐creation of research and has gained attention. To this end, the Recruitment and Retention of Alzheimer's Disease Diversity Genetic Cohorts in the Alzheimer's Disease Sequencing Project (READD‐ADSP) includes a CE Core to increase awareness of Alzheimer's disease and related dementias (ADRD) and brain health and promote participation in ADRD research across the Africa.

**Methods:**

Standard Operating Procedures based on CE principles were developed to guide equitable engagement and responsibilities for study team and Community Advisory Board (CAB) members across nine African Dementia Consortium (AfDC) countries.

Efforts included annual training on study objectives, progress reports, relevant clinical and research topics, community needs and recommendations, and goals. Partnerships were leveraged to increase brain health, dementia, and research awareness, promote recruitment and retention, and co‐create educational resources. CAB members shared expertise on their communities via focus groups (FGs) and standing meetings.

**Results:**

Established 21 CABs comprised of 141 community members across sites. Membership included community and faith leaders, public health professionals, caregivers, and NGO representatives, among others. Partnerships resulted in increased recruitment (e.g., over 50% of participants in Ibadan were from CE efforts). Co‐led community awareness campaigns and outreach efforts were conducted across study regions to maximize reach (e.g., community health screenings, educational promotion via pamphlets, social media, podcasts, caregiver video narratives, and dance classes). Following year 2 training, CAB members (*n* = 83) participated in FGs (*n* = 10). Major themes identified were prevailing misconceptions on dementia, stigma, and the need for culturally tailored information.

**Conclusion:**

Adoption of CE strategies, most notably our CAB partnerships, has ensured successful study recruitment, retention, and awareness of dementia. Co‐created resources and outreach efforts promoted a collaborative, equitable approach to brain health awareness and dementia research and in Africa. While time intensive, CE can strengthen collaborative research and community needs and priorities.

